# Early Postnatal Hemorrhagic Shock Due to Intraabdominal Hemorrhage in a Newborn with Severe Hemophilia A

**DOI:** 10.4274/Tjh.2013.0279

**Published:** 2014-03-05

**Authors:** Sara Erol, Banu Aydın, Dilek Dilli, Barış Malbora, Serdar Beken, Hasibe Gökçe Çınar, Ayşegül Zenciroğlu, Nurullah Okumuş

**Affiliations:** 1 Dr. Sami Ulus Maternity and Children Training and Research Hospital, Neonatal Intensive Care Unit, Ankara, Turkey; 2 Dr. Sami Ulus Maternity and Children Training and Research Hospital, Pediatric Hematology Unit, Ankara, Turkey; 3 Dr. Sami Ulus Maternity and Children Training and Research Hospital, Pediatric Radiology Unit, Ankara, Turkey

**Keywords:** newborn, liver, Hemorrhage, Hemophilia A, Postnatal hemorrhagic shock, Bleeding

## TO THE EDITOR

Hemophilia A, which is an X-linked recessive disorder, is one of the most common causes of congenital bleeding diathesis in newborns. Four hemophilic newborns with liver hematoma have been reported in the literature [1,2,3]. Herein, we present a newborn with hemophilia A who presented with hemorrhagic shock at the 8th hour of life subsequent to hematoma of the liver.

**Figure 1:**
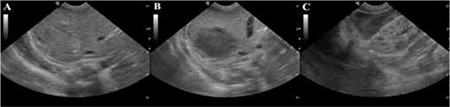
A) Hematoma located on posterior segment of the right liver lobe. B) Hypoechoic cystic lesion located on posterior segment of the right liver lobe on axial plane. C) Control ultrasonography revealed hypoechoic hematoma in the subhepatic region.

A male infant weighing 3025 g was born via normal vaginal delivery at 39 weeks. He was the first child of a 23-year-old mother. The birth was uneventful and the mother did not have any trauma history. Although the baby was normal at birth, he had severe respiratory distress at the 8th hour of life. On examination, the patient was pale and had poor capillary refill. He had bilateral cephalohematomas of about 3x2.5x2.5 cm in size in the parieto-occipital regions. Ecchymosis was noted on the scrotum and in the left inguinal regions. The parents were found to be first-degree cousins and family history revealed a maternal uncle with a diagnosis of hemophilia A. Laboratory investigation revealed a hemoglobin 8.5 g/dL, hematocrit 24.8%, leukocyte count 15000/mm3, platelet count 117000/mm^3^, the prothrombin time was 18 s, international normalized ratiowas 1.6, and activated partial thromboplastin time was 81 s. Further tests showed a factor VIII level of 0.2%. Abdominal ultrasound showed subcapsular hematoma of 40x30 mm on the posterior right lobe of the liver (Figure 1). Erythrocyte and fresh frozen plasma transfusions were made. He received an injection of vitamin K and continuous recombinant factor VIII infusion. Serial ultrasonographies showed that the size of the liver hematoma was significantly decreased. The patient was discharged from the hospital at 18 days of life and is currently on weekly recombinant factor VIII prophylaxis. Informed consent was obtained.

In severe hemophilia A (in which the level of factor VIII activity is less than 1.0% of normal), spontaneous bleeding into the joints, soft tissues, and vital organs is frequent [4]. In our patient, the factor VIII level was 0.2% when checked at the 36^th^ hour of life, and he had multiple hematomas without trauma. This situation might be due to birth trauma invaginal delivery. The optimal mode of delivery for a fetus at risk of hemophilia remains the subject of debate due to continuing uncertainty regarding the risk of intracranial and extracranial bleeding; opinions and recommendations vary [5]. 

Liver hematoma is uncommon in newborns. It has been reported in fetuses and low-birth-weight infants, and it is frequently an autopsy finding [6]. This condition is generally associated with trauma, coagulopathies, hypoxia, sepsis, maternal disease such as preeclampsia, drugs, and placental lesions [7]. 

In the previous literature, 4 patients were described with presentation of liver hematoma and all were successfully treated with factor VIII concentrates [1,2,3]. Finally, hemophilia may be present with vital organ bleeding in the newborn period. Any neonate with unexplained bleeding, and especially males, should be investigated for hemophilia. Early diagnosis and treatment can be life-saving.

## CONFLICT OF INTEREST STATEMENT

The authors of this paper have no conflicts of interest, including specific financial interests, relationships, and/or affiliations relevant to the subject matter or materials included.
